# Effect of malocclusion severity on oral health related quality of life in Malay adolescents

**DOI:** 10.1186/s12955-021-01710-2

**Published:** 2021-03-03

**Authors:** Marghana Elyaskhil, Noor Ayuni Ahmad Shafai, Norehan Mokhtar

**Affiliations:** 1 Dentistry Faculty, Kabul University of Medical Science, Ata Turk Avenue, Kabul, Afghanistan; 2 Dental Simulation and Virtual Learning Research excellence Consortium, Craniofacial and Biomaterial Sciences Cluster, Advanced Medical and Dental Institute, Universiti Sains Malaysia, 13200 Bertam, Kepala Batas, Penang Malaysia

**Keywords:** Malocclusion severity, Oral health related quality of life, Malay adolescents

## Abstract

**Background:**

The present study aims to determine the impact of malocclusion on oral health related quality of life (OHRQoL) among 13–16 years old Malay school children.

**Methods:**

School children aged between 13 and 16 years old were randomly selected from a secondary school in Penang. Malay version of Oral Health Impact Profile-14 (OHIP-14) questionnaires were given to the subjects. This questionnaire has 14 questions with seven domains which are functional limitation, psychological discomfort, physical pain, physical disability, psychological and social disability, and handicap. Index of orthodontic treatment need dental health component was used to assess the orthodontic treatment need. Overjet (reversed overjet), open bite, overbite, cross bite, impeded eruption, crowding, defects of cleft lip and palate, Class II and Class III buccal occlusion, present of supernumerary and hypodontia were assessed.

**Results:**

255 students participated in this study. Mean score and standard deviation for OHIP-14 were 8.64 (± 7.32) for males and 11.05 (± 9.41) for females respectively. There was statistically significant difference in mean score of OHIP-14 between male and female (*p* = 0.023). A weak positive correlation was found between malocclusion severity and OHRQoL (r = 0.186; *p* < 0.01). Malocclusion had a negative impact on OHRQoL of the students in the present study. This impact was prominent in psychological discomfort and psychological disability domains of OHIP-14 (*p* < 0.05).

**Conclusion:**

Increase in severity of malocclusion was associated with a negative impact on OHRQoL. Females exhibited more negative impact of malocclusion on their OHRQoL. Psychological domain was the most affected one.

## Background

Even though malocclusion is not considered as a disease and it is not a life-threatening condition, a high demand for treatment can be noticed. Malocclusion is considered an important health issue all over the world [1]. Epidemiological studies of malocclusion reported a high prevalence of this condition in different countries [2]. It seems that malocclusion and orthodontic treatment need become a health care issue and subsequently a quality of life problem [3]. The concept of oral health related quality of life (OHRQoL) is correlated with the impact of oral condition on a person’s daily function, well-being, or overall quality of life [4]. Studies on social, psychological and physical impact of malocclusion on individual’s OHRQoL lead to understanding of effects of malocclusion on people’s wellbeing.

Studies on psychological aspect of malocclusion shaded light on the effect of malocclusion and orthodontic treatment on self-esteem of adolescents [5]. Even though malocclusion can cause difficulties in maintaining oral hygiene, chewing, swallowing, speech, breathing, and predisposing to oral habits which can result in pain and discomforts, in majority of cases the key motivator for orthodontic treatment is the cosmetic impairment caused by the malocclusion [6]. Severity of the malocclusion had been found to have significant relationship with the OHRQoL, with increase in age, severe type of malocclusion leads to masticatory function deterioration and also decrease in the OHRQoL [7]. The assessment of the orthodontic treatment outcomes in patients with malocclusion highlighted a significant improvement in overall Oral Health Impact Profile-14 (OHIP-14) score [8]. While some studies declare that orthodontic treatment cannot be justified by psychological base alone [9] and that the occlusal status cannot be associated by quality of life [10].

Therefore, the need for orthodontic treatment from patient point of view can also be considered [11]. According to O'Brien et al. discrepancies can be found regarding need for treatment between patients determined by clinician using the index of orthodontic treatment need dental health component (IOTN DHC) and patient’s self-perception about their own teeth using IOTN aesthetic component (IOTN AC) [12, 13]. Many developed tools for assessing the need for orthodontic treatment are based on functional and dental health reason and they are not able to reflect the main concern of the patient which is aesthetic impairment. In Malaysia the prevalence of malocclusion in children who requires treatment is 37.4% among all ethnic groups [14]. Malocclusion can be one of the causes of dental trauma in children, Grimm et al. found a positive association between maxillary overjet and anterior teeth trauma [15]. Malocclusion can also cause conditions such as appearance discrimination, oral function problems such as jaw movement due to muscles disharmony, pain, difficulties in mastication, swallowing, speech, caries and periodontal diseases [16]. All of these factors influence the quality of life of an individual.

There is little information about impact of malocclusion on OHRQoL of school children in Malaysia. Accordingly, this study aims to determine the impact of malocclusion severity on OHRQoL among Malay adolescents.

## Materials and methods

Ethical approval was obtained from the research and human ethics committee of Universiti Sains Malaysia in May 2018. Participants in this study were school children from MARA Junior College in Penang. Student list was obtained from school, which consisted of 819 students aged between 13 and 16 years old. Subjects were recruited using simple random sampling method. Sample size was estimated based on the severity of malocclusion with 95% confidence interval and 5% margin of error therefore, the minimal number of subjects required was 231 [14]. Considering 10% attrition rate, the final sample size was 255. Students and their parents were invited and given assent and consent forms respectively to take part in the present study.

All students with their parental consents were given the Malay version of OHIP-14 questioner which has been tested for its validity and reliability by Saub et al. and asked to complete them [17]. This questionnaire has total number of 14 questions, in which it contains seven conceptual domains of OHIP-49 and each domain is represented by two questions [18]. The domains are: functional limitation, psychological discomfort, physical pain, physical disability, psychological and social disability, and handicap. After students completed the questionnaire, a IOTN DHC screening of the oral cavity was performed by a trained and calibrated examiner. Out of the 255 participants, 3 of them refused to continue with oral examination. The oral examination was performed by author in school and each student was examined for the most severe malocclusion trait using IOTN DHC. Systematic oral examination was performed according to hierarchical acronym MOCDO which stands for missing, overjet (reverse overjet), cross bites, displacement and overbite, by following this order the occlusal features were examined during subjects’ clinical assessment to determine the grade of IOTN DHC. Different occlusal traits have been graded in to five, grade, one and two represent no or little treatment need, grade three is considered as borderline for treatment need, and grade four and five are considered as definite need for treatment. Prior to data collection the examiner underwent a calibration training for the use of IOTN-DHC for a month. Intra examiner reliability was carried out on 25 subjects that were not a part of the study. The reproducibility of IOTN DHC for intra-examiner reliability was found to be very good with a Kappa score of 0.93.

The data were analysed using the Statistical Package for Social Science version 24 (SPSS,2009). Descriptive analyses, including the mean and standard deviations were performed considering the sex, age, IOTN-DHC, OHIP-14 score of students. Chi square test was performed to evaluate the association of malocclusion severity with sex. Independent sample t test was performed to assess the association between sex and students age group with OHIP-14. To evaluate the correlation between severity of malocclusion and OHRQoL Pearson correlation coefficient was performed. To assess the strength of the correlation, r > 0.50 was considered as moderate to strong correlation, and r < 0.50 indicated a weak correlation among variables. To assess the association between OHIP-14 score with age, sex and IOTN-DHC simple and multivariate linear regression analysis were performed. In simple linear regression analysis, the effect of each predictor (age, sex and IOTN-DHC) on dependent variable OHIP-14 was assessed controlling for other predictors. The selected independent variables for age, sex and IOTN-DHC. The age was stratified into 2 groups (13–14 years = 0 and 15–16 years = 1). The sex was coded as male = 0 and female = 1) and the IOTN-DHC as No need for treatment = 0, mild treatment need = 1, moderate treatment need = 2, severe treatment need = 3 and extreme treatment need = 4. In multivariate analysis the effects of multiple variables were assessed on outcome variable.

## Results

Out of 255 participants a total of 252 students were analysed in the present study. 139 (55.2%) of participants were male and 113 (44.8%) were female. 133 (52.8%) of them aged 13–14 and 119 (47.2%) were 15–16 years old. The highest percentage for the distribution of severity of malocclusion was observed for grade 3 (25.8%). Which indicates moderate need for treatment. 23.4% had grade 4 malocclusion, 22.2% of participants had grade 2, 18.7% and 9.9% had grade 1 and 5 respectively. There was no statistically significant difference in malocclusion severity between male and female participants and age groups (*p* > 0.05). Mean scores, standard deviation and observed range of the OHIP-14 and its seven domains are shown in Table 1. The mean score and standard deviation for OHIP-14 was 8.64 (7.32) for boys and 11.05 (9.41) for girls. There was statistically significant difference in mean score of OHIP-14 between male and female (*p* = 0.023). The difference was not significant among age group (*p* > 0.05) Table 2. In group comparison using One Way ANOVA followed by Bonferroni Pos Hoc test no statistically significant difference was observed in OHIP-14 score of students with different severity of malocclusion assessed by IOTN DHC (Table 3). While in group comparison of OHIP-14 domains psychological discomfort and psychological disability domains were the affected domains. In correlation analysis a weak positive relationship was observed between OHIP-14 and IOTN DHC (r = 0.186; *p* < 0.01). To evaluate the factors that affect OHIP-14 score multiple linear regression analysis was performed as in Table 4. The effect of age was not significant on overall OHIP-14 score (B = − 2.069, *p* > 0.05) (Table 5). OHIP-14 was significantly and positively associated with sex. Females had higher impact scores than males with B = 2.413 and *p* < 0.05. This association becomes stronger in multivariate model (B = 2.673; 95% CI 0.639–4.707; *p* < 0.05). It also explains about 2.1% variability among sex (r square = 0.021). There was a significant association between IOTN DHC and OHIP-14 (B = 1.235 *p* < 0.05) as well significant association was also obtained in multivariate analysis (B = 1.28; 95% CI 0.47–2.09; *p* < 0.05). According to regression model a grade increase in the IOTN DHC, will cause 1.28-fold (B = 1.28; 95% CI 0.478–2.09; *p* < 0.01) increase in overall OHIP-14 score, it also explains about 3.2% of variability (r = 0.032) among grades of IOTN DHC. In other words, the severity of malocclusion is associated with higher impact on OHRQoL.

**Table 1:** Mean, standard deviation and range observed in Oral Health Impact Profile-14 (OHIP-14)

OHIP-14 domains	Mean (SD)	Range observed
Functional limitation	0.7 ± 1.1	0–5
Physical pain	1.5 ± 1.2	0–7
Psychological discomfort	2.2 ± 1.7	0–8
Physical disability	1.4 ± 1.6	0–8
Psychological disability	1.6 ± 1.7	0–8
Social disability	0.8 ± 1.3	0–6
Handicap	1.1 ± 1.5	0–8
OHIP-total	33.6 ± 3.4	0–45

**Table 2:** Comparison of OHIP-14 score between sexes and age groups

Group	Mean (SD)	Mean difference (95% CI)	t test^a^ (df)	*p* value^a^
*Sex*				
Male	8.64 (7.32)	− 2.41 (− 4.48, − 0.33)	− 2.28 (250)	0.023*
Female	11.05 (3.39)			
*Age (year)*				
13–14	10.69 (8.45)	2.06 (− 0.05, 4.14)	1.96 (250)	0.051
15–16	8.63 (8.22)			

^a^Independent t-test

*p < 0.05 is significant

**Table 3:** Mean score and SD of OHIP-14 domains according to severity of malocclusion

IOTN DHC	Functional limitation	Physical pain	Psychological discomfort	Physical disability	Psychological disability	Social disability	Handicap	OHIP-14 total
Grade 1 (no need for treatment)	0.4 ± 0.8	1.2 ± 1.2	1.6 ± 1.4	0.9 ± 1.0	0.9 ± 1.0	0.7 ± 1.0	0.8 ± 1.1	6.8 ± 5.4
Grade 2 (mild treatment need)	0.9 ± 1.2	1.5 ± 1.1	2.0 ± 1.5	1.5 ± 1.6	1.5 ± 1.5	0.8 ± 1.2	0.8 ± 1.1	9.3 ± 7.4
Grade 3 (moderate treatment need)	0.8 ± 1.2	1.4 ± 1.4	2.2 ± 1.8	1.5 ± 1.7	1.8 ± 1.8	0.8 ± 1.5	1.1 ± 1.6	10.5 ± 10.3
Grade 4 (severe treatment need)	0.5 ± 0.8	1.7 ± 1.2	2.6 ± 2.0*	1.5 ± 1.6	2.0 ± 1.9*	0.9 ± 1.3	1.4 ± 1.8	11.0 ± 8.5
Grade 5 (extreme treatment need)	1.2 ± 1.4	1.5 ± 1.0	2.8 ± 1.5*	2.0 ± 1.8	2.1 ± 1.7	0.9 ± 1.4	0.9 ± 1.4	12.2 ± 9.1

Group comparisons were performed by One Way Analysis of Variance (ANOVA) and Bonferoni Post Hoc test

^*^P < 0.05

**Table 4:** Multivariate linear regression model showing association of sex and IOTN-DHC with OHIP-14

Dependent variable	Independent variable	B	t	95% CI	*p* value
OHIP-14	Sex (female)	2.67	2.58	0.639–4.70	0.010
	IOTN-DHC	1.28	3.13	0.47–2.09	< 0.001

Dependent variable: OHIP-14

**Table 5:** Simple linear regression model showing association of age, sex and IOTN-DHC with OHIP-14 score

Independent variable	R^2^	B	t	95% CI	*p* value
Age (15–16 years)	0.015	− 2.069	− 1.965	− 4.143 to 0.05	0.05
Sex (female)	0.021	2.413	2.288	0.336–4.489	0.02
IOTN-DHC	0.034	1.235	2.975	0.418–2.053	0.03

Dependent variable: OHIP-14

Reference category for age: 13–14 years; reference category for sex: male

## Discussion

This study assessed the impact of malocclusion on overall OHRQoL and its domains in Malay schoolchildren aged 13–16 years old. Regression analysis indicated that severity of malocclusion has an impact on OHRQoL and increase in severity of malocclusion leads to poor OHRQoL. These findings are in line with previous studies [8, 11, 12, 19]. Similar result was obtained from multivariate regression analysis, increase in severity of malocclusion was associated with increase in overall score of OHIP-14. OHRQoL was assessed better in teens and increase in age was correspondent with poor OHRQoL. This result is not supported by the present study as the sample are students aged 13–16 years old.

The impact of malocclusion on OHRQoL was found to be significant. Similar result was obtained by Chen et al. their findings indicated that severity of malocclusion was associated with greater impact on OHRQoL. This study was performed on a sample of 18–25 years old patients. Malocclusion assessment was carried out by IOTN-DHC and OHRQoL was evaluated by the OHIP-14 measure. Patients who were in borderline and high orthodontic treatment need showed higher score in OHIP-14 scale compared to those with no need for orthodontic treatment [20]. Age group was negatively associated with overall OHIP-14 score. Masood et al., reported that participants aged between 15 and 18 years old showed the highest impact of malocclusion on OHRQoL [11]. Evaluation of the impact of malocclusion on different domains of OHIP-14 showed that the greatest effect was observed for psychological discomfort domain. In multivariate regression analysis, the effect of severe type of malocclusion was dominant on OHRQoL Participants with little, borderline and high orthodontic treatment need showed 5, 9 and 15 points higher score on OHIP-14 than those with “no treatment need” respectively.

Assessment of malocclusion with different measures such as dental aesthetic index (DAI) showed similar results to the present study. Malocclusion in anterior segment found to be negatively affected by the OHRQoL and schoolchildren with anterior segment spacing, midline diastema and overjet exhibited 30% more negative impact than those without malocclusion [8]. However, these malocclusion traits do not indicate malocclusion in this age. It seems that effects of malocclusion become prominent in early stages of life on OHRQoL.

Despite of different age groups and instruments used for assessment of the malocclusion and OHRQoL there is an agreement on the impact of malocclusion on OHRQoL. Malocclusion severity, socioeconomic status and clinical conditions contributes with poor OHRQoL [7]. The impact of racial and sociodemographic variables also reported [21]. In the present study the study sample consisted of Malay ethnic group. This could be one of the limitation of present study. Different ethnic groups have different norm for aesthetic therefore inclusion of other ethnic groups could better represent the Malaysian population. This study also did not evaluate the severity of malocclusion according to IOTN-AC, this could add the need for orthodontic treatment from prospective of the students.

## Conclusion

Malocclusion was associated with a negative impact on OHRQoL of the students in the present study. The negative impact was dominant in psychological discomfort and psychological disability domains. This study provided information that low quality of life is associated with severity of malocclusion.

## Data Availability

All data has been provided in the manuscript.

## Abbreviations

OHRQoL: Oral health related quality of life
OHIP: Oral Health Impact Profile
IOTN DHC: Index of orthodontic treatment need dental health component
IOTN AC: Index of orthodontic treatment need aesthetic component
MOCDO: Missing, overjet (reverse overjet), cross bites, displacement and overbite
DAI: Dental Aesthetic Index
